# Diversity of fruit-feeding butterflies in a mountaintop archipelago of rainforest

**DOI:** 10.1371/journal.pone.0180007

**Published:** 2017-06-30

**Authors:** Geanne Carla Novais Pereira, Marcel Serra Coelho, Marina do Vale Beirão, Rodrigo Fagundes Braga, Geraldo Wilson Fernandes

**Affiliations:** 1 Universidade Federal de Minas Gerais/Ecologia Evolutiva & Biodiversidade/DBG, ICB/, Belo Horizonte MG, Brazil; 2 Universidade Estadual Paulista, Instituto de Biociências, Departamento de Botânica, Laboratório de Fenologia, Rio Claro, São Paulo, Brazil; 3 Universidade Federal de Ouro Preto, Programa de Ecologia de Biomas Tropicais, Campus Morro do Cruzeiro, Ouro Preto, Minas Gerais, Brazil; 4 Universidade Federal de Lavras, Setor de Ecologia e Conservação, Campus Universitário, Lavras, Minas Gerais, Brazil; 5 Universidade do Estado de Minas Gerais, UEMG, Unidade Divinópolis, Divinópolis, MG, Brazil; Charles University, CZECH REPUBLIC

## Abstract

We provide the first description of the effects of local vegetation and landscape structure on the fruit-feeding butterfly community of a natural archipelago of montane rainforest islands in the *Serra do Espinhaço*, southeastern Brazil. Butterflies were collected with bait traps in eleven forest islands through both dry and rainy seasons for two consecutive years. The influence of local and landscape parameters and seasonality on butterfly species richness, abundance and composition were analyzed. We also examined the partitioning and decomposition of temporal and spatial beta diversity. Five hundred and twelve fruit-feeding butterflies belonging to thirty-four species were recorded. Butterfly species richness and abundance were higher on islands with greater canopy openness in the dry season. On the other hand, islands with greater understory coverage hosted higher species richness in the rainy season. Instead, the butterfly species richness was higher with lower understory coverage in the dry season. Butterfly abundance was not influenced by understory cover. The landscape metrics of area and isolation had no effect on species richness and abundance. The composition of butterfly communities in the forest islands was not randomly structured. The butterfly communities were dependent on local and landscape effects, and the mechanism of turnover was the main source of variation in β diversity. The preservation of this mountain rainforest island complex is vital for the maintenance of fruit-feeding butterfly community; one island does not reflect the diversity found in the whole archipelago.

## Introduction

Mechanisms that maintain the structure of communities have aroused great interest from the scientific community [1]. Environmental conditions can play a percussive role, or filters, facilitating or hindering the establishment of species [2,3]. Environmental parameters, indicative of environmental structure, function as a filter in the structuring of communities, preventing the establishment of some species [4,5]. These filters can be global (e.g. effects of climate), regional (e.g., effects of landscape) and local (e.g., effects of habitat). Ecological relationships that are antagonistic (e.g., predation, herbivory, parasitism, competition) or mutualistic (pollination, facilitation), may represent the most important structuring mechanisms of biological communities, having the potential to influence the distribution of species along environmental gradients [5].

Fruit-feeding butterflies are excellent models for testing hypotheses regarding the effects of environmental filters in tropical biological communities. Their use is facilitated by the availability of relatively simple and cheap trapping methods that permit replication and standardization of sampling for comparisons among different environments, besides being taxonomically well known [6]. Among local factors that affect tropical forest butterfly communities, the frequency and intensity of clearings, microclimate (e.g., temperature, wind and rain), luminosity, presence of host plants for caterpillars and food resources for adults are of great relevance (e.g. [7–9]). These factors are strongly related to vegetation structure [10], which is a component of essential habitat for butterflies given its relationship to thermoregulation and the provision of resting and mating locations [11,12]. Forest butterfly communities are also influenced by structural factors of the habitat, such as topography, vertical stratification, edge effect, matrix quality as different levels of disturbance or even matrix of natural grasslands [13–16]. Two variables can be easily measured to characterize local or habitat structure: openness of the canopy and understory cover. Canopy openness is related to the level of humidity and temperature; the more dense or closed the canopy, the less solar incidence and the more humid becomes the habitat [8]. Often, species richness and abundance of insects tend to be greater in forest habitats with more open canopies (e.g. [8,17]), including butterflies [18], however some gaps resulted by anthropic impacts can cause opposite effects [19]. The density or coverage of understory vegetation is another important habitat variable that influence community structure. High density of understory vegetation can hinder the foraging and reproductive activities of butterflies, resulting in a decline in species richness (e.g. [20,21]).

Regional factors affecting butterfly communities in tropical forests include features of the landscape that influence the entire system via edge effects [22]. Area and degree of isolation of forest fragments are important metrics for insect communities. Area can influence resource abundance and the structural complexity of vegetation [10,23–25]. Therefore, species richness can be positively correlated with area and negatively with isolation [26–28]. However, this topic is not completely understood for invertebrates. There are correlations of increased (e.g. [23,29,30], decreased (e.g. [31]) and absence of effect (e.g. [32,33]) between species richness of invertebrates and area. The species richness can decline with increased isolation in simple landscapes and small fragments, but not in complex landscapes and large fragments [34]. Species richness is determined by a balance between area or isolation, wherein the number of species tends to become constant over time due to the continuous process of replacement of species or *turnover* [26,35–37]. Some butterflies have large thorax volumes combined with comparatively shorter forewing lengths allowing long flights. Specially those butterflies with adaptive morphologies permit foraging flights under a regional scale [24]. It is also expected that species richness of forest specialists increase with increasing connectivity among fragments, as well as with the area of adjacent forest fragments, as already documented for butterflies (see [38]). Large fragments or continuous areas of forest can function as a continent in a source-sink system and the closest fragments as stepping stones (see [39]**)**, facilitating the movement of species among fragments in a possible metacommunity dynamic (see [40]).

Local and regional environmental parameters also act synergistically to produce and maintain patterns of diversity, both in space and time. Habitat structure also has a strong influence on patterns of beta diversity of arthropods [41]. Beta diversity refers to the diversity among habitats, and the difference in species composition among locations or intervals of time [42]. This difference can be explained by the substitution of species (turnover) or the loss of species (nesting) depending on the intensity of local and regional forces acting on the community [43]. Responses to habitat heterogeneity vary among butterfly subfamilies according to the foraging habitats and adaptations [19]. Seasonality is also recognized as a strong driver of butterfly communities. Seasonality is very determinant to plant phenology, and butterfly communities follow those circles of resources availability [44,45].

Naturally fragmented landscapes represent an important scenario where one can observe the forces that shape community structure [33]. Although widely studied in islands (e.g. [32,46]) and artificially fragmented areas (e.g. [47–51]), there are few studies on butterflies in naturally fragmented environments [52,53]. It is likely that different processes lead to different patterns in islands with natural vegetation. Natural fragmented forests can be found in the highest points (above 1200 meters in elevation) in the Espinhaço mountain range in southeastern Brazil, immersed in rupestrian grassland vegetation matrix [16]. These islands of rainforest vegetation require specific climate and soil to develop, being found in erosion valleys, in locations without boulders, covering hills, and forms a true natural archipelago of forest vegetation strongly associated with the regions of headwaters, rivers, creeks and small streams [16,54,55].

The patterns of diversity of fruit-feeding butterfly communities were analyzed in an archipelago of natural islands of Atlantic rainforest in the Espinhaço mountain range, Brazil. In this study we tested the following hypotheses: i) the openness of the canopy has a positive effect on the richness and abundance of butterflies; ii) the coverage of the understory has a negative effect on the richness and abundance of butterflies; iii) the richness and abundance of butterflies are greater in the rainy season than in the dry season; iv) the richness and abundance of fruit-feeding butterflies increases with the size of forest islands (e.g. area and perimeter of island); v) the richness and abundance of fruit-feeding butterflies decreases with level of isolation; vi) the species composition of fruit-feeding butterflies of the forest islands is not structured by chance; vii) the species composition of fruit-feeding butterflies is dependent on both local (openness of canopy and understory cover) and landscape (area and isolation) effects and seasonality.

## Materials and methods

### Study location

**Fig 1:**
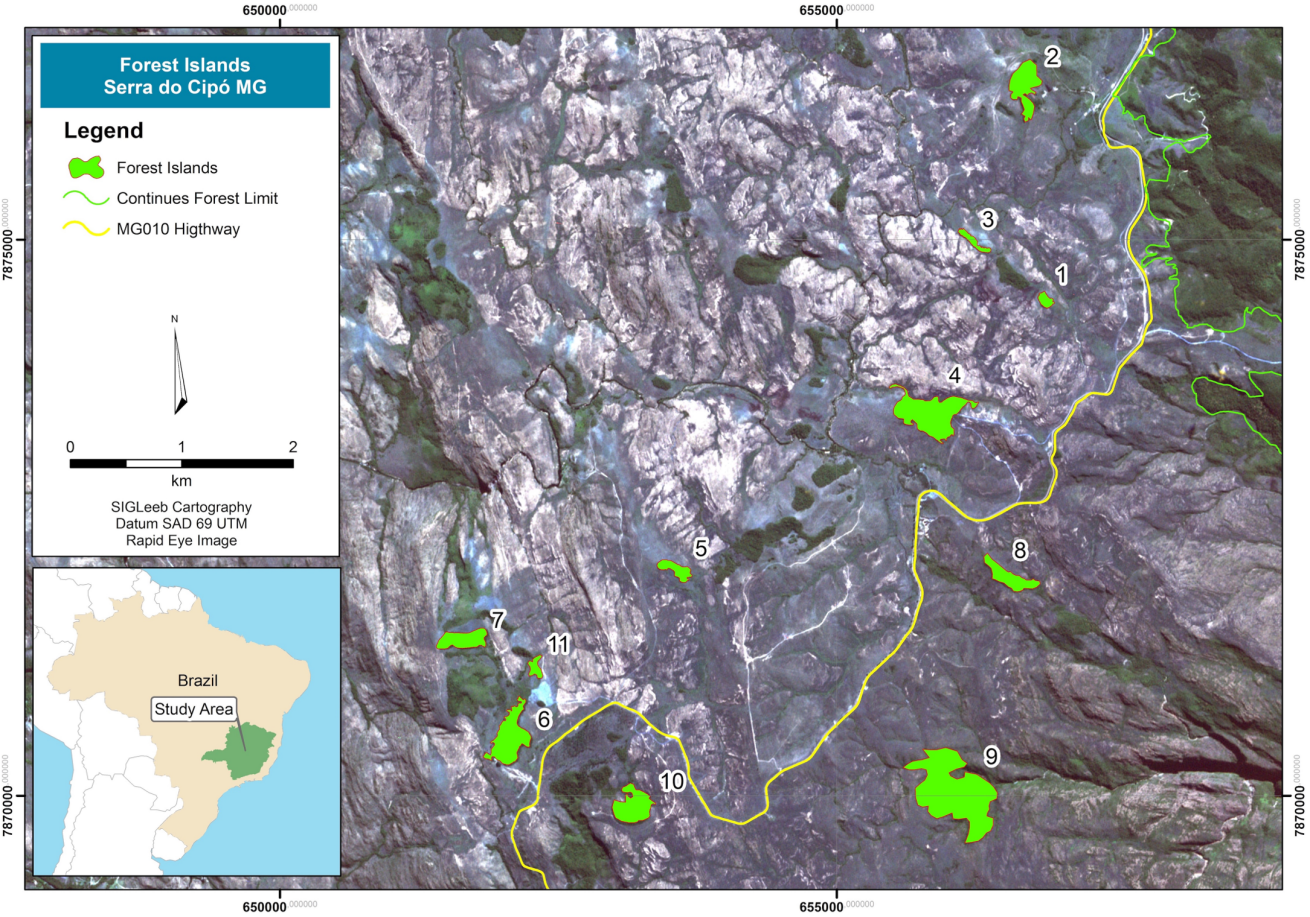
Map of the study area, Serra do Cipó, Minas Gerais, Brazil (SIGLeeb Cartografy Rapid Eye Image 2015).

The study area was located in *Serra do Cipó*, municipality of *Santana do Riacho*, *Minas Gerais*, Brazil (19°14'19"S 43°31'35"W, Fig 1), in the southern portion of the Espinhaço mountain range. The climate is type Cwb (mesothermal according to the Köppen classification) with humid summers and dry winters. Average annual rainfall ranges from 1,300 to 1,600 mm and is concentrated in the summer, and average temperatures are between 17.4 and 19.8°C [56]. Floristically, forested islands found in *Serra do Cipó* are associated with the Atlantic Forest domain, with some *Cerrado* species [16]. The elevation of the studied islands varied from 1230 to 1331 meters above sea level. The average annual temperature of the region for 2014 and 2015 was 18.8°C (data from meteorological stations Onset HOBO^®^ U30 data-logger, installed at 1200, 1300 and 1400 meters of altitude in the study site).

### Sampling

#### Sample design

**Fig 2:**
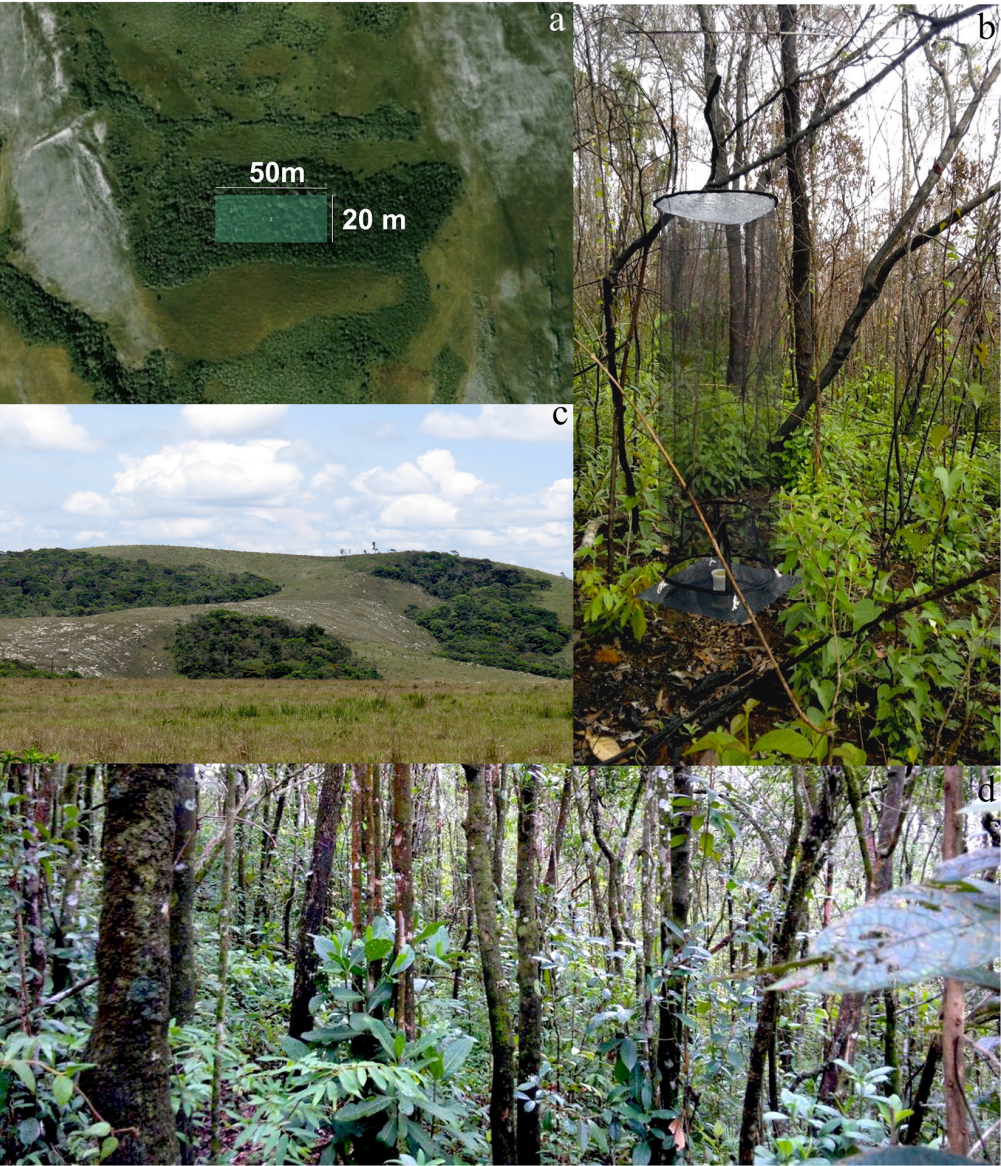
(A) Schematic drawing of 20x50m plot established on the islands (Image @ 2015 CNES/Astrium- Google Earth Pro). (B) Van Someren-Rydon frugivorous butterfly trap. (C): External view of the islands 5. (D) Internal view of the island.

Eleven rain forest islands of different sizes were selected (Fig 1). Three islands were located within the *Parque Nacional da Serra do Cipó* (Islands 8, 9 and 10) while the remainder were located within the buffering park zone named *Área de Proteção Ambiental Morro da Pedreira* (hereafter APA) (Table 1) [16]. Islands were chosen considering their size (large enough so that part could be sampled for butterflies), state of conservation (preference for islands with low anthropic impact) and accessibility (Fig 2A, 2B, 2C and 2D). The studied islands varied in size from 12,938 m^2^ to 358,185 m^2^ (Table 1). In each island, a 50 x 20 m plot was established at least 20 m from the border, except for Islands 3, 5, 8 and 11, which were closer to the edge due to their small size and shape (Fig 2A). To observe the effect of seasonality on variation in butterflies, two samples were made per year, one during the rainy season (February) and the other during the dry season (August) for the two consecutive years of 2014 and 2015.

**Table 1:** Area, perimeter, distance to nearest continuous forest, distance to closest forest island, altitude, location, and geographic coordinates of the 11 forest islands in Serra do Cipó, Brazil.

Island #	Area (m^2^)	Perimeter (m)	Distance to the continuous forest (km)	Distance to closest forest island (km)	Altitude (m)	Location	Coordinates	
1	12,938	480	0.88	0.13	1,239	APA	S 19 13' 01.97237''	W 43 30' 28.67035''
2	84,909	1,807	1.14	0.56	1,235	APA	S 19 11' 58.18605''	W 43 30' 31.58434''
3	16,316	857	1.4	0.11	1,234	APA	S 19 12' 47.65259''	W 43 30' 46.21099''
4	169,562	2,836	2.06	0.78	1,269	APA	S 19 13' 34.60436''	W 43 30' 55.97316''
5	29,716	911	5.47	0.23	1,309	APA	S 19 14' 21.24191''	W 43 32' 26.10892''
6	113,399	2,220	7.37	0.19	1,317	APA	S 19 15' 10.76660''	W 43 33' 07.45422''
7	58,653	1,192	7.35	0.1	1,331	APA	S 19 14' 40.91360''	W 43 33' 20.64709''
8	57,557	1,366	2.49	0.34	1,271	Park	S 19 14' 19.32289''	W 43 30' 45.68173''
9	358,185	3,685	3.5	0.3	1,230	Park	S 19 15' 18.03651''	W 43 31' 01.00603''
10	82,375	1,502	6.2	0.21	1,324	Park	S 19 15' 34.38313''	W 43 32' 32.32418''
11	16,113	675	6.91	0.3	1,273	APA	S 19 14' 52.58462''	W 43 33' 03.19909''

#### Butterfly collection

At each corner of each plot a Van Someren-Rydon butterfly trap was set (n = 4 traps/island; n total = 44) (Fig 2B). The traps consisted of a 110 cm tall and 35 cm diameter fine screen cylinder that was closed at the top. The base of the trap consisted of a platform on which a 50 ml plastic cup with bait was placed [57]. Traps were baited with fermented banana with sugar cane juice at a ratio of 3:1, which was prepared two days prior to use, and suspended between 70 and 100 cm above the ground. Butterflies attracted by the smell of the bait entered through an opening in the bottom of the cylinder to feed, and when they moved upward they would become trapped [58]. Each field campaign consisted of five sampling days, the first to set the traps and the following four to collect the sampled individuals and change bait every 24 hours. The total effort was 704 days-traps (44 traps x 4 samplings x 4 sampling days). Collected individuals were sacrificed by thoracic compression and placed in entomological envelopes with sampling data, including date, island number and trap number, for later identification. In the laboratory, individuals were identified to the lowest possible taxonomic level using guides [59–61] and the help of taxonomists. After identification, three individuals of each species (whenever possible) were mounted, correctly prepared and deposited in the *Laboratório de Ecologia Evolutiva & Biodiversidade*, of the *Universidade Federal de Minas Gerais*.

#### Canopy openness

To evaluate canopy openness of islands, hemispherical photos were taken with a fisheye lens attached to a Pentax digital camera from each of the four corners of each plot of each island. The photos were taken at 1.50 m above the ground and then processed for the proportion of white and black pixels, which were averaged over the four photos to get a single value of canopy openness for each island. These data were collected during all periods of butterfly sampling in both seasons. The images were processed using the R software package “ReadImages” [62] and “RT4Bio” [63].

#### Understory cover

Digital images of shrubby and herbaceous vegetation were used to measure the influence of understory vegetation cover on the butterfly community. The vegetation was photographed at the corner of each plot using a 100 cm x 100 cm white screen as a backdrop with the camera positioned at three meters from the screen and one meter above the ground [20,64]. Four photos of the understory, one in each cardinal direction, were taken at each corner of the plots for a total of 16 photos for each island for each campaign. The areas of the white backdrop were cut from the photos and edges added to define the part of the photo to be analyzed using the software Gimp 2 (Gnu Image Manipulation Program 2.8.14). Very dark photos with patches of shadows or sun were discarded. Vegetation cover is defined as the ratio of white and black pixels in the photos [20]. The average of the values of the photos taken at each measurement were used as a value for the understory cover for each island. The images were processed using the R software package “EBImage” [62].

#### Landscape parameters

The distance of each island to the continuous forest and the distance to other islands were used as a measure of isolation of the forest islands [39]. The metrics of area, perimeter and distance between fragments was obtained with help of the software FRAGSTATS [65]. After vectorization, the file was converted into a format compatible to FRAGSTATS (ASCII) [66]. The distances between forest islands were generated from the automated definition of the analyzed centroids.

### Statistical analyses

The richness estimator Jackknife1 was used to estimate species richness, a nonparametric method that utilizes the number of rare species this is found in a single sample (uniques) [67].

Due to the nature of the data, two categories of models were designed to test local (vegetation parameters according to seasonality) and regional influences on the butterfly community. All of the statistical analyses were performed using the software R [68].

To test the effect of vegetation depending on the seasonality on butterfly species richness and abundance, the butterfly community values were used as response variables (species richness and abundance), while the interaction between vegetation variables (canopy openness and understory coverage) and seasons (dry and rainy) were used as explanatory variables. The forested islands were included as random factors in a Generalized Linear Mixed Model (GLMM) [69], using the “*Poisson*” distribution of errors for the two variables (richness and abundance). The analysis was conducted using the package “lme4” [70], with the function “glmer”. All of the variables were tested together and the non-significant ones (p > 0.05) were removed (stepwise) until the minimum model adequacy is obtained. The package “MuMIn” [71] was used with the function “r.squaredGLMM” [72] to obtain the value of R^2^. To visualize the effect of the significant variables using graphs we performed separate Generalized Linear Models (GLMs). The Generalized Linear Models were conducted in order to allow the graphical interpretation of the Generalized Linear Mixed Model results.

To test the hypothesis that fruit-feeding butterfly species richness and abundance increases with island size and decreases with island isolation, the butterfly community metrics (richness and abundance of species accumulated in all samplings) were used as response variables and the variables of the landscape (area and perimeter of each island, distance to the closest island and to continuous forest) were used as explanatory variables in a Generalized Linear Model (GLM) [69] with a “*Quasipoisson*” distribution of errors for both richness and abundance. To test the diversity between the season, fruit-feeding butterfly species richness and abundance per sample were used as response variable and the season were used as explanatory variable in a Generalized Linear Model (GLM) [69] with a “*Quasipoisson*” distribution of errors for both richness and abundance.

To test what regional scale most contributes to the total number of species in all islands an analysis of additive partition of diversity (β = γ- α) was used. The alpha (α) represents the diversity of each island, the beta (β) represents diversity between islands, and the gamma (γ) represents total diversity of the forest archipelago. The packages “vegan” [73] and “betapart” [74] were used with the functions “adipart” [75], “beta.pair” and “beta.temp” [76]. The difference of the diversity between the forest islands (spatial β) or along the sampling time (temporal β) can be due to nestedness (species gain or loses) or turnover (species replacement among the forest islands or sampling time) of species. An analysis of β partition was made to test if the temporal and spatial β are due to turnover or nestedness. This analysis was made using the package “vegan” [73] and results in three indexes: the Simpson index (βSIM) expresses the turnover, the Søresen index (βSOR) expresses the total β, and nesting is expressed by the Søresen index minus the Simpson index (β_SNE_) [76].

To test the dependence of local, landscape and seasonal effects on butterfly species composition, Permutational Mutlivariate Analysis of Variance (PERMANOVA) [77] was performed using the package “vegan’ [73] and the function “adonis”. For this analysis, the butterfly community composition was used as the response variable and the landscape variables (perimeter and area of the island, distance to the closest island and to continuous forest), vegetation variables (canopy openness and understory coverage) and seasonality (dry and rainy) were used as the explanatory variables.

## Results

A total of 512 fruit-feeding butterflies individuals of 34 species of the Nymphalidae family, belonging to seven tribes and three subfamilies Biblidinae (4 individuals), Caraxinae (5 individual) and Satyrinae (504 individuals) were collected (Table 2). The most abundant species were *Godartiana muscosa* (Satyrinae) with 189 individuals (36.8%), followed by *Yphithimoides straminea* (Satyrinae) with 69 individuals (13.4%) and *Moneuptychia soter* (Satyrinae) with 44 individuals (8.6%). Of all the 34 species, 15 were singletons (representing 44.1% of the species). None of the species occurred on all of the islands. Species richness varied from 3 (Island 3) to 18 species (Island 5) while the abundance varied from 4 (Island 6) to 100 individuals (Island 10) (Table 2). Based on the Jacknife 1 richness estimator, 68.4% of the species present were collected (estimated 49.6 species).

**Table 2:** Frequency, richness and singletons of species of frugivorous butterflies collected in the archipelago of forest islands in Serra do Cipó, Brazil. Abundance of butterfly subfamily is shown within parentheses.

*Species*	Island #											Season		Total
	1	2	3	4	5	6	7	8	9	10	11	Wet	Dry	
**Biblidinae** (4)												** **	** **	** **
**Tribe Biblidini**														
*Hamadryas feronia* (Linnaeus, 1758)									1				1	1
**Tribe Callicorini**												** **	** **	** **
*Callicore sorana* (Godart, [1824])								1				1		1
**Tribe Catonephelini**														
*Catonephele acontius* (Linnaeus, 1771)		1										1		1
*Myscelia orsis* (Drury, 1782)		1										1		1
**Charaxinae** (5)														
**Tribe Preponini**														
*Archaeoprepona demophoon* (Hübner, [1814])				2			1					3		3
**Tribe Anaeini**														
*Fountainea ryphea* (Cramer, 1775)								1					1	1
*Memphis moruus* (Fabrícius, 1775)		1										1		1
**Satyrinae** (503)														
**Tribe Brassolini**														
*Blepolenis batea* (Hübner, [1821])		1	2		2		3	4	1			13		13
*Caligo arisbe* (Hübner, [1822])				13			5	4	1	6	4	33		33
*Dasyophthalma rusina* (Godart, [1824])		1		1				1				3		3
*Eryphanis automedon* (Cramer, 1775)				3					1			3	1	4
*Opoptera syme* (Hübner, [1821])							4	4	12	5		25		25
*Opsiphanes invirae* (Hübner, [1808])									1			1		1
**Tribe Satirini**														
*Carminda griseldis*						1						1		1
*Erichthodes narapa* (Schaus, 1902)					1								1	1
*Forsterinaria necys* (Godart, [1824])					1				10	1		7	5	12
*Forsterinaria quantius* (Godart, [1824])				9				1	12	7		10	19	29
*Godartiana muscosa* (A. Butler, 1870)	6		8	6	22	1	23	34	13	65	11	71	118	189
*Hermeuptychia* sp.			3		9		4					11	5	16
*Moneuptychia itapeva* (Freitas, 2007)			2		5		1	1				4	5	9
*Moneuptychia soter* (A. Butler, 1877)	1		1		15	1	20			5	1	11	33	44
*Paryphthimoides eous* (A. Butler, 1867)					1							1		1
*Paryphthimoides phronius* (Godart, [1824])					13		3						16	16
*Paryphthimoides poltys* (Prittwitz, 1865)					3		1	1				3	2	5
*Pharneuptychia* sp.					2	1	1				1	3	2	5
*Pharneuptychia phares* (Godart, [1824])					1							1		1
*Praepedaliodes phanias* (Hewitson, 1862)									1			1		1
*Yphthimoides angularis* (A. Butler, 1867)	1		1		1		9				2	10	4	14
*Yphthimoides manasses* (C. Felder & R. Felder, 1867)											1		1	1
*Yphthimoides ochracea* (A. Butler, 1867)					2			1					3	3
*Yphthimoides pacta* (Weymer, 1911)					1								1	1
*Yphthimoides straminea* (A. Butler, 1867)			2	1	1		3	35	10	10	7	51	18	69
*Yphthimoides yphthim*a (C. Felder & R. Felder, 1867)					1				1	1		2	1	3
*Zischkaia pronophila* (Butler, 1867)		3										1	2	3
**Abundance**	8	8	19	35	82	4	78	88	64	100	27	273	239	512
**Richness**	3	6	9	8	16	4	14	17	13	10	11	27	20	43
**Singletons**		3			5	1		2	3		1	9	6	15

### Local effects

**Fig 3:**
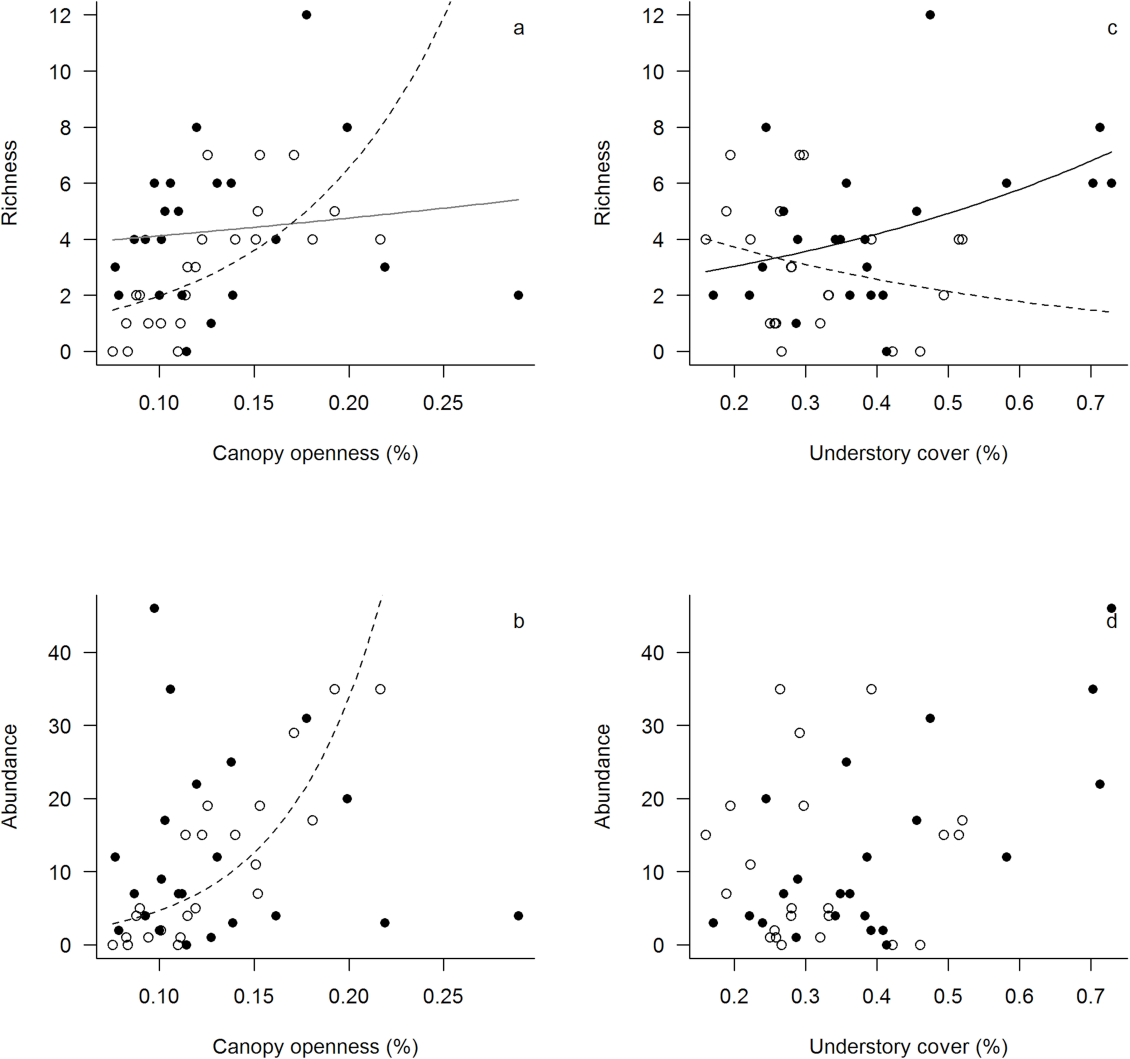
Effect of canopy openness and understory coverage on richness and abundance of frugivorous butterflies by season (dry and rainy), based on simplified generalized linear mixed model (Table 3). (A) Richness with canopy openness in the dry season—p < 0.001; (B) Abundance with the canopy openness in the dry season—p < 0.001; (C) Richness of butterflies with understory coverage in the rainy season—p = 0.03; (D) Abundance of butterflies with understory coverage in the dry season—p = 0.02. (D) Legend: dry season—dashed lines and empty circles, rainy season—solid lines and full circles.

Canopy openness of the forest islands varied from 7% (island 14 in the dry season) to 29% (island 10 in the rainy season), while the average canopy openness was 12.87% (±4.5SE). The effect of canopy openness on butterfly richness and abundance was different depending on the season. Canopy openness did not affect the richness and abundance of butterflies in the rainy season. In the dry season, richness (p < 0.001) and abundance (p < 0.001) of butterfly species were greater in islands with greater canopy openness (Fig 3A and 3B; Table 3).

**Table 3:** Analyses of the models showing the effect of canopy openness, understory coverage and seasonality on the richness and abundance of frugivorous butterflies of a montane forest archipelago in Serra do Cipó, Brazil (richness R^2^ = 53.1 p<0.001; abundance R^2^ = 83 p<0.001).

	Richness		Abundance	
	F	p	F	p
**Canopy openness (%)**	0.266	0.521	8.211	< 0.001 *
**Understory cover (%)**	**6.772**	0.029 *	15.489	0.266
**Season (Dry and Wet)**	0.685	0.646	0.711	< 0.001 *
Interaction canopy * season	5.315	0.022 *	**57.388**	< 0.001 *
Interaction Understory * season	5.310	0.024*	3.425	0.066

In bold the values with the best explanation F_4,39_.

* indicates a statistically significant relationship.

Understory coverage varied from 16% (island 9 in the dry season, ±0.022SE) to 73% (island 10 in the rainy season, ±0.034SE), while the average understory coverage was 35.8% (±0.021SE). The effect of understory coverage on butterfly richness was dependent on the season (Table 3). In the rainy season, richness of butterflies was greater in islands with higher understory coverage (p = 0.03); while in the dry season, richness of butterflies was greater in islands with lower understory coverage (p = 0.02) (Fig 3C). The abundance of butterflies was not affected by the understory coverage in any season (p = 0.3) (Fig 3D).

**Fig 4:**
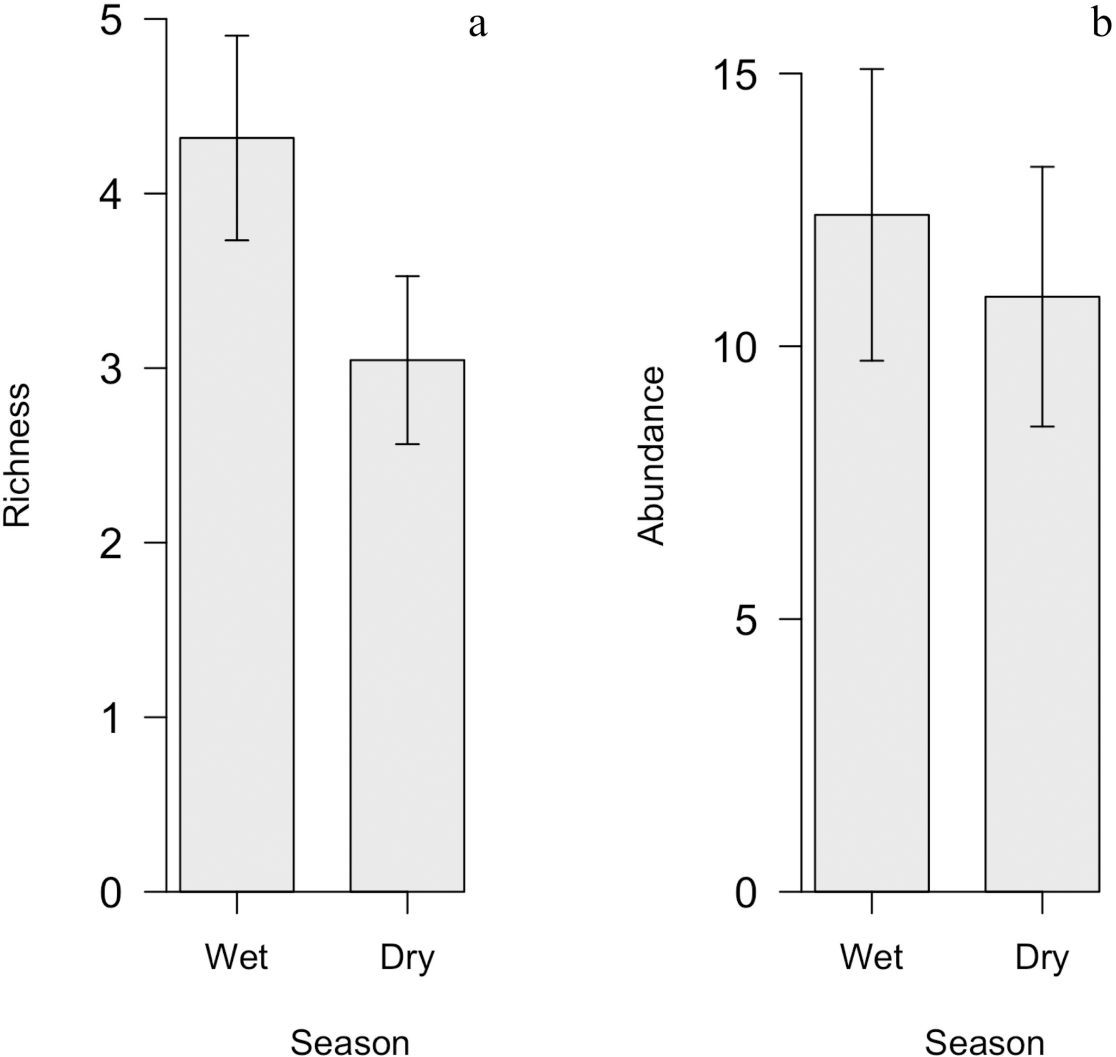
Richness and abundance of frugivorous butterflies in each season (dry and rainy) by forest island, based on simplified generalized linear mixed model. (A) Richness in the rainy and dry seasons. (B) Abundance in the rainy and dry season. There was no significant difference between the two seasons.

Fruit-feeding butterfly richness did not vary with season (Fig 4A). Of all the 34 species collected, 27 species were collected in the rainy season and 20 species in the dry season. The average number of species of butterflies per island was 3.68 (SE = 0.83), wherein 4.3 (SE = 0.58) in the rainy season and 3.04 (SE = 0.48) in the dry season. Of all the 34 species sampled 13 species (38.2%) occurred in both season, 14 (41.2%) were exclusive of the rainy season and seven (20.6%) of the dry season. There was no significant difference in butterfly abundance between the rainy and dry seasons. The average abundance of butterflies per island was 11.66 (±2.53 SE) individuals, with no significant differences between seasons in the simplified model (Fig 4B).

### Landscape effects

The landscape metrics (area, perimeter, distance to closest island and to continuous forest) did not influence the richness or the abundance of fruit-feeding butterflies of the forest islands of the *Serra do Cipó* (richness F_3,6_ = 2.03; p = 0.22; abundance, F_3,6_ = 1.71; p = 0.28).

### Species composition

**Fig 5:**
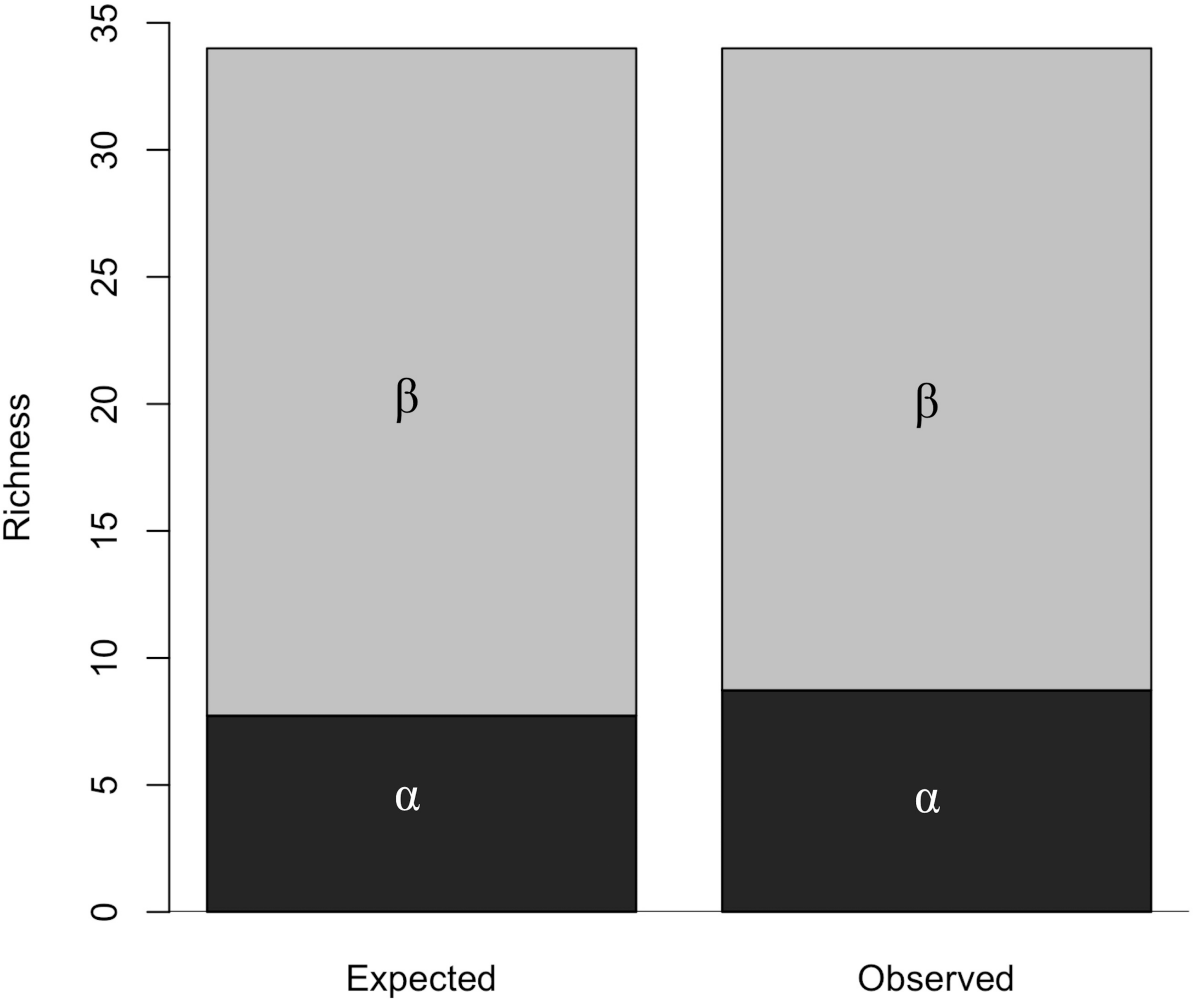
Observed and expected diversity of frugivorous butterflies of a montane forest archipelago in Serra do Cipó, Brazil. Observed alpha diversity was greater than expected. Observed beta diversity was less than expected.

The partitioning of diversity showed that α (forest island diversity) was responsible for 25.6% of the total diversity (average of 8.7 species), a greater diversity than expected if individuals were distributed at random (expected = 21.6%; α = 7.36; p < 0.001; Fig 5). The contribution of β diversity contribution (diversity among islands) was responsible for 74.3% of the total diversity (average of 25.3 species) and was less than expected by chance (expected = 78.3%; β = 26.6; p < 0.001). Nevertheless, β diversity was the diversity scale that most contributed to the total diversity of the forest archipelago (α = 25.6% and β = 74.3%). The difference in beta diversity among islands was caused mainly by the process of species turnover (75.9% of β, β_SIM_ = 0.49), while the processes of nesting explained only 24.1% of the beta diversity (β_SNE_ = 0.15).

The temporal partition of β indicated that the turnover was responsible for 78.3% of the total β diversity among islands in time (β_SOR_ = 0.518; β_SIM_ = 0.406), while nesting represented 21.7% (β_SNE_ = 0.112), indicating that the community of butterflies showed distinct phenological cycles.

Fruit-feeding butterfly composition of the forest islands varied with area, season and canopy openness (R^2^ = 23.6; Table 4). Perimeter, understory coverage, distance to the closer island and distance to continuous forest did not significantly influence the species composition of butterflies in the studied forest islands.

**Table 4:** Variables that significantly influence the species composition of frugivorous butterflies of a montane forest archipelago in Serra do Cipó, Brazil.

Variables	F	R^2^	p_value_
Area	2.01	0.085	0.02 *
Season	1.85	0.078	0.03 *
Canopy openness	1.71	0.079	0.04 *

Values of F, R^2^ and p for variables tested (Permanova; R^2^ 0.236 total).

* indicates a statistically significant relationship.

## Discussion

Mountain forest archipelagos are still poorly known and the ecological process are just beginning to be investigated [16]. Our study suggests that naturally fragmented forested mountaintops host lower richness compared to other types of fragmented forests in Brazil [38,78]. According to this lower species richness, the abundance of each species was also lower (15 singletons) making the diversity in each island different from the others (high beta diversity due to turnover). Species abundance was also lower in our study (15 singletons), resulting in considerable variation in diversity across islands (high beta diversity due to turnover).

The genus with the greatest representation of species was *Yphthimoides* (6 species and 91 individuals), which is very diverse in Atlantic rain forest areas and open areas of *Cerrado* (savanna) in central Brazil [79] and has both forest and matrix specialist species [38]. In areas of Atlantic rain forest under the process of fragmentation, the abundance of forest specialist species is concentrated in October to March (rainy season), while the specialist species of the matrix are concentrated from July to September (dry season) [38]. This variation is due to different foraging, reproduction and predator avoidance strategies that are related to reproductive success [38,44]. Some species of *Yphthimoides*, as well as other species found in the forest islands in the dry season, may come from the surrounding matrix of Rupestrian Grasslands with the forest islands serving as ecological refuges for these species.

The environmental conditions of the forest islands are more favorable than those of the matrix in the dry season. Rupestrian grasslands have high temperatures and low humidity in the dry season (see [56]). A similar pattern occurs in seasonal deciduous forest, where the greatest abundance of butterflies in open areas occurs due to the great abundance of the butterflies adapted to the strong solar exposure. However, rupestrian grasslands are characterized by high temperatures during the dry season, when this positive relation no longer occurs [17]. A great density of habitat generalist species in small fragments reflects the nature of the surrounding landscape [80]. The various responses of insects to fragmented environments are dependent on the surrounding matrix, with influences on migration or isolation of the species among fragments [34,52,81]. Some species found in the forest islands in the dry season were more abundant in habitats with greater canopy openness because they have natural resistance to low levels of humidity, high temperatures and strong solar incidence (e.g. *F*. *ryphea* and *H*. *feronia*; [38]).

Canopy openness positively influenced butterfly richness, abundance (in the dry season) and species composition, revealing an important structural factor of the community of the fruit-feeding butterflies in the mountain forest habitats studied. Canopy variation can play a very important role in the diversity of butterflies [6,15,82,83]. In a Peruvian rain forest, the effect of fall-tree gaps caused an increase in fruit-feeding butterfly because it created a mosaic of habitats [83]. The greater butterfly richness and abundance observed in islands with more open canopies in the dry season may also be related to the large representation of the subfamily Satyrinae and by the transition between the borders of the forest islands and the matrix of rupestrian grasslands. These islands host species of both forest and open environments. Transition environments formed by a gradient of forest to ruderal grasses can house a larger Satyrinae fauna, whereas a greater abundance is expected in the grassland (see [13,21,38]. At the borders of fragments, as well as in clearings, there is intense regeneration and growth of plants capable of maintaining a large number of species and diversity of butterflies [13]. Research involving forest clearings points to changes in butterfly species composition in ecotonal environments [83,84], resulting in a greater diversity of butterflies [83]. Considering that fragments are strongly influenced by edge effects (see [22]), a great number of species of the subfamily Satyrinae were expected to occur in the study islands [13,85].

Butterfly richness was higher in islands with greater understory coverage in the rainy season, yet higher in islands with lower understory coverage in the dry season. The abundance of butterfly was not significant different in islands with lower understory coverage in the dry season. Many satyrs have a low flight pattern [86] and high understory coverage may obstruct the movement of some insect species [20]. For example, very dense vegetation alters the escape behavior of grasshoppers, which end up falling to the ground or making very long flights, exposing themselves to predation [20]. Some leafhoppers (sub-order Auchenorrhyncha) are also negatively affected by high density of vegetative cover [87]. A study conducted in different areas of central Europe recorded structural heterogeneity of the understory as the most important indicator in explaining increases in butterfly species richness [88]. Structural heterogeneity of the understory tends to increase in edges and where the canopy is not structured, promoting a more illuminated environment, and thus, more resources for butterflies, such as host plants and fruit, for example [88]. However, an environment with greater understory coverage may not be more heterogeneous because it may be dominated by a limited number of plant species. When comparing fragmented, restored, and pasture areas a negative correlation between butterfly richness and density of herbaceous vegetation were found [21]. Our results seem to reveal a trade-off between two fundamental environmental conditions of butterfly’s communities: food availability and mobility. Throughout the rainy season—reproductive season—food availability seems to explain the higher butterfly richness whereas the mobility seems to stand out as the predominant driver in the dry season when the resources are not abundant and the foraging movement is fundamental.

Despite that the annual reproductive cycle of the frugivorous butterflies generally follows the phenological cycle of their host plants and variation in temperature, our results did not show any statistical difference in butterfly richness and abundance between seasons [45]. Neotropical butterfly communities normally are expected to be more abundant and richer in the rainy season in response to the greater availability of resources [6]. A larger number of species were found in the rainy season in our study. The composition also varied according to the season (27 species in the rainy season, 20 in the dry one and 13 in both seasons), however these differences were not significant. Of the exclusive species, nine were *singletons* in the rainy season and six in the dry season. That is, in addition hosting more species, the rainy season also hosts rarer species (less abundant). Seven species were collected only in the dry season: *H*. *feronia*, *F*. *ryphea*, *P*. *phonius*, *Y*. *manasses*, *Y*. *ochracea*, *Y*. *pacta* e *E*. *narapa*. *F*. *ryphea* (Charaxinae). The most of the species of Charaxinae subfamily disperse very well and the family is very representative in open areas of *Cerrado* [53]. The tribe Brassolini (79 individuals) occurs predominantly in the rainy season, and we collected six species during that time: *B*. *batea* (13), *C*. *arisbe* (33), *D*. *rusina* (3), *O*. *syme* (25), *O*. *invirae* (1) and *E*. *automedon*. Only *E*. *automedon* (1 individual) was collected in the dry season. This tribe is the most abundant in continuous forest rather than fragmented landscape [78] and in the interior of the fragment of Atlantic Forest [89], but is also strongly correlated with the vegetation mosaic in Atlantic Forest [7]. Individuals of the tribe Brassolini were found in almost all of the islands except in two (Islands 1 and 6), indicating that this tribe is well represented in the forest islands of *Serra do Cipó*. Brassolini caterpillars feed on Poaceae and Arecaceae [90], and species from both plant families occur within the islands [16]. The absence of the expected pattern—i.e. difference in butterfly richness and abundance between seasons—might be from utilization of forest island resources of butterfly species coming from the matrix composed by rupestrian grasslands. Butterflies from grasslands could use the forest island to escape from harsh conditions of the dry season, when the average temperature increases and humidity decreases.

Our hypothesis that species richness and abundance of fruit-feeding butterflies increases with island size and reduces with island isolation was not corroborated, as seen in other studies [29]. In fragments in the Amazon, the number of species of butterflies did not increase with area [48] as predicted by “niche theory” and “related species-sorting models”. The niche theory assumes that differences in the responses of species to resources are not similar and that their spatial distributions are controlled by ecological conditions. The same predictions drive the modern metacommunity models [39,40]. The relationship of species diversity of butterflies and area in an anthropic fragmented environment in Germany was dependent on the degree of species specialization. In a study of fragments of Atlantic Forest, there was no correlation of richness and abundance with area, suggesting that the surrounding matrix is more important as a determinant of the butterfly fauna than fragment size [13,21,38]. In a naturally fragmented area, [53] also reported that the matrix was not a barrier to the movement of some species of fruit-feeding butterflies, but that type of matrix could influence the isolation of fragments [52]. The literature shows that the increase in butterfly richness with increasing size of fragments was more closely related to the availability of host plants than with fragment size [78]. The influence of area and isolation of fragments is dependent on how specialized is the species [38]. Forest specialist species are found in greater numbers in fragments with larger areas and higher levels of connectivity, while species tolerant of matrix habitats experience increased abundance with decreasing forest cover [38]. Thus, our data indicates that island size had an influence only on species composition.

Environmental heterogeneity among the forest islands was an important predictor of butterfly species composition [91]. Habitat heterogeneity strongly influences the patterns of beta diversity of fruit-feeding butterflies, as well as other arthropods [41]. The factors influencing the composition of fruit-feeding butterflies in the archipelago of forest islands were: seasonality, canopy openness and island area. The β diversity contributes the most for the total diversity and mostly due to turnover of species. β diversity was less than expected by chance. The scale of diversity that most contributed to the total composition was the β diversity and the turnover process the main source of variation. In other words, each island in the forest archipelago has an important role in the total diversity of the whole archipelago. The high turnover rate of species between islands suggests that the local scale has a fundamental role in structuring the butterfly community. Other works also support the importance of the local scale for communities of butterflies [7,51], caterpillars [41] and other arthropods [41]. The data reported here show the importance of each forest island in maintaining the structure and conserving the communities of the fruit-feeding butterflies associated with the entire mountainous forest archipelago.

## Final considerations

The results reported here indicate that species richness and abundance of fruit-feeding butterflies was higher on islands with greater canopy openness and that this pattern is dependent on seasonality. Fruit-feeding butterfly richness is dependent of understory coverage and seasonality. The results also demonstrated that the landscape metrics (e.g. area, perimeter and island isolation) had little effect on richness and abundance of fruit-feeding butterflies of the studied area, but strongly influenced species composition. Turnover is the main source of variation in β diversity, probably due to the high number of singleton species (44% of the composition). Thus, local habitat characteristics were more important than those of the landscape in structuring the butterfly communities of the forest archipelago associated with rupestrian grassland of the *Serra do Cipó*. Preserving this island complex is of great importance for maintaining the diversity of fruit-feeding butterflies. One single island separate from the archipelago does not harbor the diversity and process found through the entire system.
